# Adult-Onset Pediatric-Type Follicular Lymphoma of the Parotid Gland

**DOI:** 10.7759/cureus.76161

**Published:** 2024-12-21

**Authors:** Mazin J Albaldawy, Tamer Ebaied, Humaid O Bin Harmal Al Shamsi, Ashraf Nadaf, Rahil U Faruk Abbu

**Affiliations:** 1 Department of Oncology, Al Dhannah Hospital, Al Dhannah, ARE; 2 Department of ENT (Ear, Nose and Throat), Al Dhannah Hospital, Al Dhannah, ARE; 3 Medical Oncology Department, Burjeel Medical City, Abu Dhabi, ARE; 4 Department of Radiology, Al Dhannah Hospital, Al Dhannah, ARE; 5 Biomedical Sciences, University of Chicago, Chicago, USA

**Keywords:** b-cell lymphoma, marginal zone lymphona, parotid gland tumor, pediatric-type follicular lymphoma, rare benign tumor

## Abstract

Pediatric-type follicular lymphoma (PTFL) is an extremely rare B-cell lymphoma that primarily affects children and young adults, typically in individuals under 25 years old, with a median age of 15 years. Here, we report a rare case of PTFL in a 27-year-old adult male who presented with a slow-growing mass near his left ear. Initial CT scans of the neck revealed two oval-shaped, smooth, well-defined, homogeneously enhancing soft tissue density lesions in the superficial lobe of the left parotid gland. A complete surgical excision was performed, and a postoperative ultrasound of the neck confirmed complete removal, with only hypoechoic changes observed in the parotid gland. Comprehensive immunohistochemical analysis showed CD20-positive B cells co-expressing germinal center markers CD10 and BCL6 but negative for BCL2 and cyclin D1. The Ki-67 proliferation index was notably elevated, indicating high cellular activity. Additional findings included MEF2B positivity, confirming the lymphoma diagnosis, and an immunoglobulin gene rearrangement, which demonstrated a monoclonal B-cell population consistent with a neoplastic process. CD21 staining further revealed distorted follicular dendritic cell networks and attenuated IgD-positive mantle. Fluorescence in situ hybridization (FISH) analysis showed no rearrangements in BCL2, IRF4, or BCL6, and any deletion in the 1p36 region (TNFRSF14 gene), ruling out other lymphoma types. Histologically, the nodules showed distorted secondary follicles with obscured germinal centers, confirming PTFL. At the three-month follow-up, the patient demonstrated satisfactory healing with no signs of recurrence. This case underscores the importance for oncologists to perform a thorough differential diagnosis of head and neck masses, as PTFL can present with characteristics similar to classical follicular lymphoma, IRF4-rearranged large B-cell lymphoma, pediatric nodal marginal zone lymphoma, and reactive follicular hyperplasia. A comprehensive diagnostic approach, including clinical, pathological, and immunohistochemical analyses, is essential for developing an accurate diagnosis and management plan for PTFL, especially in atypical adult presentations.

## Introduction

Pediatric-type follicular lymphoma (PTFL) is a relatively rare B-cell lymphoma that primarily affects children and young adults. It often appears with regional nodal illness, which frequently involves the head and neck regions [1]. Even though follicular lymphoma is one of the most prevalent lymphoid malignancies in adults, it can also affect young adults, though less frequently (1-2%) [2,3].

Recognized as a distinct subtype in the revised 2016 World Health Organization Lymphoma Classification, PTFL shares morphologic and phenotypic similarities with adult-type follicular lymphoma and pediatric nodal marginal zone lymphoma [4]. PTFL is more frequently associated with grade 3 morphology, and a significant proportion of patients present with stage 1 disease at diagnosis [5].

Management of PTFL typically involves complete surgical excision followed by observation. Consistent with PTFL treatment approaches, the United States National Comprehensive Cancer Network (NCCN) guidelines generally advocate for a watch-and-wait strategy for benign tumors, moving away from chemotherapy as a first-line treatment. However, the NCCN still suggests considering radiotherapy or chemotherapy for affected regions in cases of severe local disease [6]. Children and adolescents with follicular lymphoma have a good prognosis with either limited treatment or a "watch and wait" approach after total resection [7,8]. With a five-year survival rate of 95% and a two-year disease-free survival rate of about 94% following treatment, patients with advanced-stage PTFL generally have excellent prognoses [8].

## Case presentation

Initial consultation and symptoms

A 27-year-old male patient initially consulted an ENT specialist with complaints of an isolated neck mass and swelling in the left parotid gland. The patient reported that the mass had been present for three months, during which time it had gradually increased in size. The mass was painless without any inflammatory features. He had no recent history of illness or fever as well. Given the nature of the symptoms and the persistence of the mass, the case was eventually referred to oncology for further evaluation and management. The physical examination revealed that the lump was 5 cm in size and was growing upward. No concomitant symptoms, including fever, exhaustion, sweats at night, weight loss, or pain, were reported by the patient.

Computed tomography (CT) scan

A series of CT scans were conducted, including scans of the neck, thorax, abdomen, and pelvis. These imaging studies were crucial in assessing the anatomical structures, identifying any abnormalities, and determining the presence or extent of mass involved.

**Figure 1:**
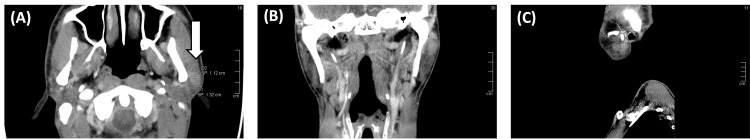
Multiregional CT imaging of neck, thorax, abdomen, and pelvis showing normal structures with isolated parotid gland lesions (A) CT neck scan showing two oval-shaped, well-defined, homogeneously enhancing soft tissue lesions in the superficial lobe of the left parotid gland, measuring 1.12 cm × 1.32 cm (arrow). (B) CT thorax scan with normal lung volume and attenuation and no focal lesions in mediastinal and thoracic structures. (C) CT abdomen and pelvis scan showing normal liver, biliary system, and other abdominal organs, with no abnormalities.

The CT neck scan (Figure 1A) revealed two oval-shaped, smooth, well-defined, homogeneously enhancing soft tissue density lesions in the superficial lobe of the left parotid gland, measuring 1.31 cm × 1.32 cm. The remainder of the parotid gland, along with the oropharynx, nasopharynx, hypopharynx, vocal cords, salivary glands, and thyroid gland, appeared normal, with no mass lesions or abnormal cervical lymphadenopathy observed. Additionally, the cervical spine was found to be normal, showing no fractures or dislocations. The CT thorax scan (Figure 1B) demonstrated that both lungs were normal in volume and attenuation, with no focal lesions identified. The mediastinal structures showed no obvious lymphadenopathy, indicating a lack of significant disease involvement in this area. Additionally, the cardiac and vascular structures, including the heart, aortic arch, thoracic aorta, and other great vessels, appeared normal. The rib cage and thoracic spine also exhibited no significant abnormalities, further supporting the absence of thoracic complications related to the patient's condition. The CT abdomen and pelvis scan (Figure 1C) revealed that the liver and biliary system were of normal size and contour, with no mass lesions detected. The intrahepatic biliary and portal radicals were also found to be normal. Furthermore, other abdominal organs, including the gallbladder, spleen, pancreas, kidneys, and urinary bladder, were reported as normal, with no abnormalities noted in any of these structures. This comprehensive assessment indicates a lack of significant findings in the abdominal and pelvic regions.

Biopsy

**Figure 2:**
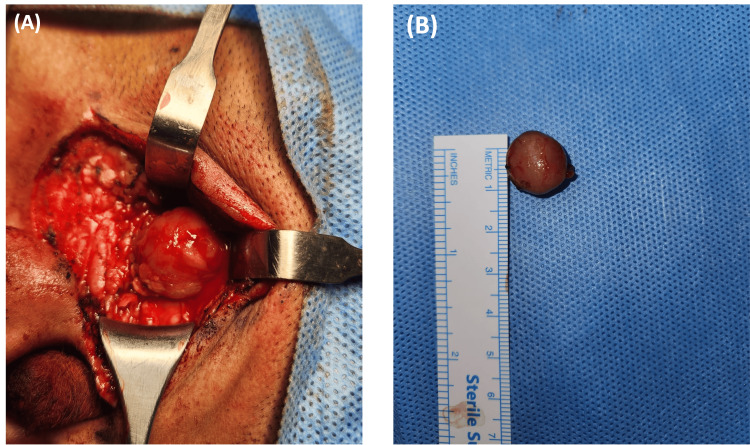
Intraoperative and post-excision photographs of parotid tumor removal (A) Intraoperative photograph showing the left-sided formal lazy-S parotid incision used to access the superficial parotid region, providing clear exposure of the tumor while preserving the left facial nerve. (B) Post-excision image of the excised, well-defined circular mass placed beside a ruler, indicating an approximate size of 1 cm in length.

The surgical procedure, as shown in Figure 2A, involved a left-sided formal lazy-S parotid incision, followed by dissection in the superficial parotid region, with careful preservation of the left facial nerve. This incision provided full exposure of the mass, which had a well-defined boundary and was not attached to the surrounding tissue. The tumor was successfully and completely excised (Figure 2B).

Ultrasound

**Figure 3:**
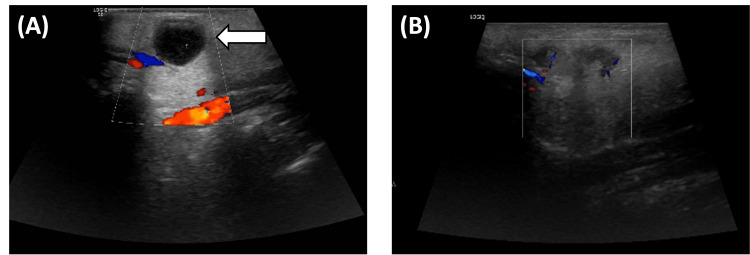
Ultrasound evaluation of the left parotid region pre and post-biopsy (A) Pre-biopsy ultrasound image of the left parotid gland, showing a well-defined, vascularized lesion within the superficial lobe (arrow). (B) Post-biopsy ultrasound image demonstrating postoperative changes, with a hypoechoic area at the site of the excised lesion. No residual or recurrent mass lesions are observed, and surrounding soft tissues in the neck region appear unremarkable, indicating no significant postoperative complications.

Following the successful surgical excision of the parotid mass, ultrasound imaging was performed to assess the neck region. As seen in Figure 3A, the pre-biopsy ultrasound demonstrates a well-defined, vascularized lesion within the superficial lobe of the left parotid gland, which was subsequently confirmed to be the excised mass. In the post-biopsy ultrasound (Figure 3B), postoperative changes are evident, characterized by a hypoechoic area in the region where the lesion was previously located. No residual or recurrent mass lesions were observed, and the remaining soft tissues in the neck appeared unremarkable, indicating an absence of any significant postoperative complications or abnormalities.

Pathology and molecular diagnosis 

Following the surgical excision, immunohistochemical and molecular pathology tests were undertaken to identify PTFL from other lymphoproliferative or neoplastic tumors. Given the complexity of the case and the need for additional diagnostic workup not available at Union 71/PureHealth, the case was referred to Mayo Clinic Laboratories - Rochester Main Campus for a comprehensive evaluation and diagnostic confirmation.

Immunohistochemistry

Immunohistochemical studies revealed that CD20-positive B cells were co-expressing germinal center markers such as CD10, BCL6, and MEF2B while being negative for BCL2 and cyclin D1. Additionally, the CD21 stain highlighted distorted and expanded follicular dendritic cell networks underlying these atypical nodules. The high Ki-67 proliferation index, along with the lack of polarization, further supported the neoplastic nature of the growth. The specimen also tested negative for CD30, confirming the absence of activated B-cell markers. Kappa light chain restriction within the atypical lymphoid nodules confirmed the presence of a monoclonal B-cell population. Critical stains were repeated to ensure diagnostic accuracy, with CD20/PAX5-positive B cells predominantly observed in the enlarged atypical nodules, which co-expressed germinal center markers. These nodules also displayed attenuated IgD-positive mantle zones surrounding them.

Histology

**Figure 4:**
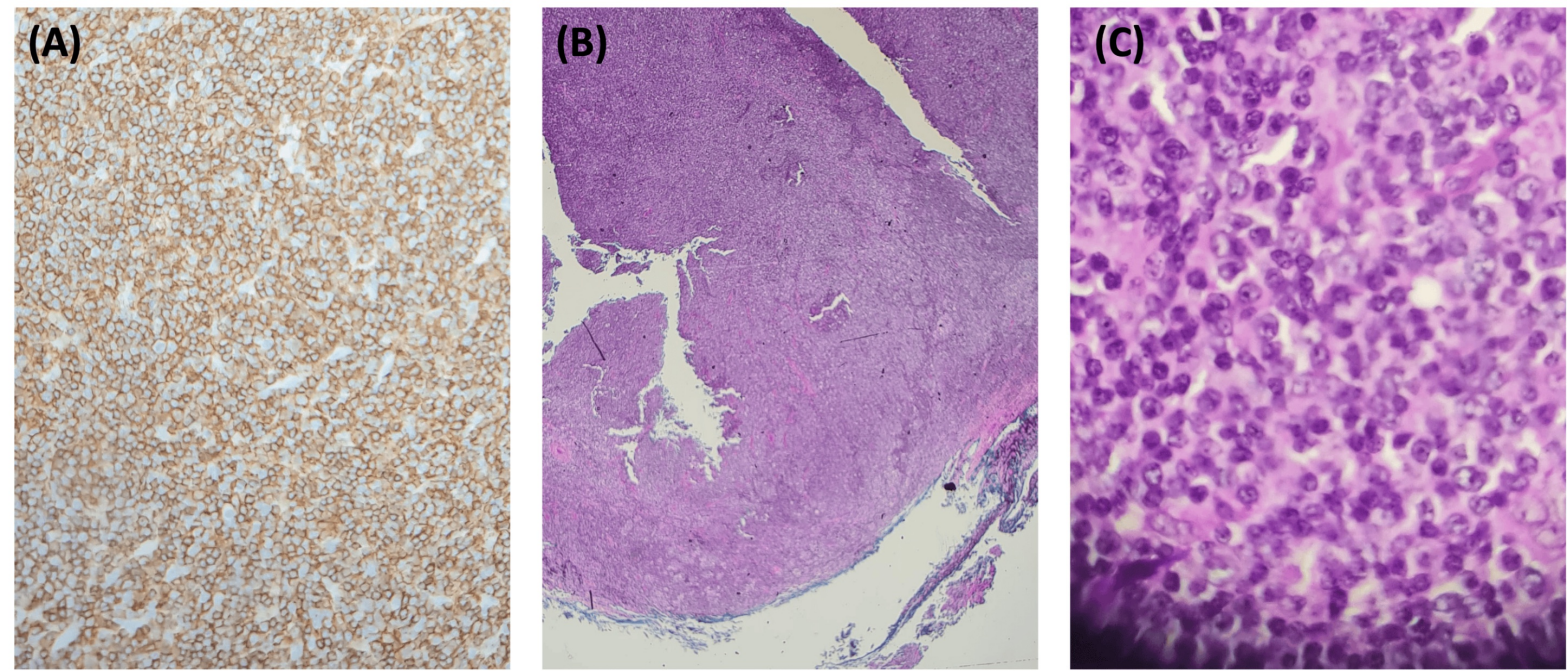
Microscopic imaging of parotid gland tumor with CD20 and hematoxylin and eosin staining (A) Immunohistochemical staining for CD20 at 10× magnification demonstrates a predominance of CD20-positive B cells within the enlarged and distorted atypical nodules, indicating a monoclonal B-cell population. (B) Hematoxylin and eosin staining at low magnification (40×) reveals nodular architecture with follicular hyperplasia, highlighting abnormal lymphoid proliferation and distorted follicle structures. (C) Hematoxylin and eosin staining at high magnification (80×) displays atypical lymphoid cells with prominent nucleoli and dense chromatin, supporting a neoplastic process.

The histological examination of the left parotid gland specimen revealed an enlarged lymph node with partial nodal effacement characterized by an atypical nodular proliferation of distorted secondary follicles. As seen in Figure 4B-4C, the follicles exhibited expanded and obscured germinal centers, lacking definitive polarization, and were associated with pale lymphoid areas suggestive of marginal zone differentiation. The microscopic observation of CD20-positive B cells showed enlarged and distorted atypical nodules (Figure 4A). At the periphery, reactive-appearing secondary follicles suggested mild reactive hyperplasia, and the presence of an atypical lymphoid infiltrate in a portion of the surrounding salivary gland tissue added to the pathological findings.

Immunoglobulin Gene Rearrangement Test

The immunoglobulin gene rearrangement analysis performed on the excised tumor yielded a positive result for clonal immunoglobulin gene rearrangement. This indicated the presence of a monoclonal population of B cells, which is consistent with a neoplastic process, such as lymphoma.

B-cell Lymphoma FISH Test

Fluorescence in situ hybridization (FISH) analysis of the left parotid tissue showed normal results for all loci studied, with no rearrangements of IRF4, BCL2, or BCL6. Additionally, no deletion of the 1p36 chromosome region, where the TNFRSF14 gene is located, was observed. The absence of these common lymphoma-associated genetic alterations is typical of PTFL and helped to exclude other subtypes.

Follow up

Six weeks after the tumor was surgically removed, a positron emission tomography (PET) scan was performed to look for any remaining tumor or abnormal cells in the left parotid gland. The PET scan indicated no evidence of regional spread or distant disease. The patient was also scheduled for evaluations every three months for two years, including clinical testing, ultrasound imaging, and PET scans, to monitor for any recurrence of the cancer.

## Discussion

Despite histological similarities, PTFL in adults is exceptionally rare, and these patients generally experience favorable outcomes following surgical intervention. Thus, for our case, a multidisciplinary approach that included imaging, histological, and immunohistochemical evaluations was conducted to make a definite diagnosis.

CT scans and ultrasound were employed to assess the disease's extent and the surgical intervention's effectiveness. The CT scans of the neck, thorax, abdomen, and pelvis provided essential insights into the localization and potential staging of the disease. The CT scan of the neck (Figure 1A) revealed two well-defined lesions in the left parotid gland, raising suspicion of residual lymph nodal lesions. The smooth and homogeneous enhancement of these lesions suggested that they were likely residual rather than new or unrelated pathology. Additionally, the absence of significant abnormalities in the surrounding structures and lymph nodes was a positive sign, suggesting that the disease might have been localized rather than widespread. In the thoracic evaluation, the CT scan of the thorax (Figure 1B) showed normal findings, with no evidence of pulmonary involvement or mediastinal lymphadenopathy. This was favorable for lymphoma staging, as it suggested that the lymphoma might have been confined to the neck region without systemic spread to the thoracic cavity. Similarly, the CT scan of the abdomen and pelvis (Figure 1C) demonstrated normal findings, with no evidence of lymphoma involvement in these regions. The absence of any mass lesions or lymphadenopathy in the abdominal cavity was a crucial positive indicator for staging the disease, reinforcing the likelihood that the patient's lymphoma might have been localized. Ultrasound imaging was also performed to assess the neck region and confirm the characteristics of the parotid lesion. The pre-biopsy ultrasound (Figure 3A) revealed a well-defined, vascularized lesion within the superficial lobe of the left parotid gland. These imaging findings align with existing literature, highlighting the localized nature of PTFL, primarily affecting the head and neck region with minimal extranodal involvement [9,10]. Notably, our patient’s imaging results corroborate the observations of Louissaint et al., who reported that PTFL rarely exhibits systemic dissemination or involvement of distal lymph nodes [9].

Surgical management is a cornerstone of PTFL treatment, with studies by Liu et al. reporting excellent outcomes following excision, with no recurrence observed during long-term follow-up [10]. Thus, given the findings from the imaging, surgical resection of the mass in the left parotid gland was considered the most appropriate treatment approach. The surgical procedure, as shown in Figure 3A, involved a left-sided formal lazy-S parotid incision, followed by dissection in the superficial parotid region, allowing for complete and successful excision of the tumor. This was further confirmed by the post-biopsy ultrasound (Figure 3B), which showed the absence of visible mass lesions.

Subsequently, a series of laboratory investigations were conducted to confirm the diagnosis of PTFL and further characterize the tumor's molecular and pathological features. The positive result for clonal immunoglobulin gene rearrangement in the left parotid gland specimen was a crucial finding indicative of a B-cell lymphoproliferative disorder [11]. This result was obtained through a PCR-based assay targeting the heavy and kappa light chain genes, specifically designed to detect rearrangements typical of neoplastic B-cell processes. The B-cell lymphoma FISH test was also conducted to detect chromosomal abnormalities. The FISH analysis yielded normal results for all loci studied, including the IRF4, BCL2, and BCL6 genes, as well as the 1p36 chromosome region where the TNFRSF14 gene is located [12]. The absence of rearrangements in these genes was particularly relevant, as alterations in these regions are typically observed in more aggressive forms of B-cell lymphoma, such as follicular lymphoma or diffuse large B-cell lymphoma [13]. The lack of these chromosomal alterations, alongside the positive immunoglobulin gene rearrangement, suggested that while a clonal B-cell population was present, the absence of high-risk genetic abnormalities might indicate a less aggressive disease course. These findings were consistent with the diagnosis of PTFL, which is known to have a more indolent and less aggressive clinical course than other B-cell lymphomas [10]. The study by Louissaint et al. further supports this, where it was showcased that PTFL typically lacks the genetic mutations commonly found in adult follicular lymphoma, reinforcing its indolent behavior [9].

The expanded immunohistochemical panel further validated the diagnosis of PTFL. The analysis demonstrated that CD20-positive B cells (Figure 4A) co-expressed CD10 and BCL6, which are germinal center markers commonly associated with follicular lymphoma [14]. Notably, the B cells in the atypical nodules were negative for BCL2 and cyclin D1, crucial in distinguishing PTFL from adult-type follicular lymphoma [1]. The elevated Ki-67 proliferation index and the absence of the typical polarization seen in benign conditions like follicular hyperplasia provided further support for the diagnosis of PTFL [1]. Previous reports have similarly highlighted the negative BCL2 expression as a defining feature of PTFL [9,10]. Additionally, the elevated Ki-67 index and disrupted follicular architecture observed in this case were consistent with findings from Louissaint et al. and Liu et al., providing further evidence for the diagnosis of PTFL [9,10]. The MEF2B positivity, a marker typically associated with lymphoma rather than reactive hyperplasia [15], confirmed the neoplastic nature of the lesion. Histological examination of the specimen showed distorted secondary follicles with expanded germinal centers and marginal zone differentiation (Figure 4B-4C). The observed distortion in follicular architecture, characterized by attenuated IgD-positive mantle zones and disrupted follicular dendritic cell networks, was highly suggestive of PTFL [15]. These structural changes, together with the immunohistochemical and molecular data, reinforced the diagnosis and helped differentiate PTFL from other lymphoma subtypes.

Six weeks after surgery, a PET scan was conducted to evaluate for any remaining tumor or abnormal cells. The scan revealed no evidence of regional spread or distant disease, confirming that the patient was in remission. In accordance with PTFL management protocols, the patient was scheduled for follow-up evaluations every three months for two years, including clinical assessments, ultrasound imaging, and PET scans, to monitor for potential recurrence. This approach aligns with the NCCN guidelines, which recommend a "watch-and-wait" strategy following complete surgical excision of benign tumors like PTFL, emphasizing the avoidance of chemotherapy as a first-line treatment [6].

In summary, we were able to distinctly diagnose PTFL from other lymphoid conditions by integrating key histological, immunohistochemical, and molecular features. Follicular hyperplasia was excluded due to its lack of clonal B-cell populations, preserved follicular architecture, and absence of an elevated Ki-67 proliferation index. Pediatric nodal marginal zone lymphoma was ruled out as it typically shows marginal zone differentiation and does not express the germinal center markers (CD10 and BCL6 positivity) observed in our case. IRF4-rearranged large B-cell lymphoma was excluded because it is associated with IRF4 translocations, which were not present in this case. The immunohistochemical findings, including CD20-positive, CD10-positive, BCL6-positive, BCL2-negative, and cyclin D1-negative markers, along with MEF2B positivity and atypical histology marked by distorted follicular architecture and attenuated IgD-positive mantle zones, were crucial in confirming the diagnosis of PTFL. Additionally, the absence of chromosomal rearrangements commonly associated with aggressive lymphomas, such as BCL2, BCL6, and IRF4, further supported the diagnosis. Given PTFL's indolent clinical course and its rare presentation in adults, our thorough multidisciplinary diagnostic approach enabled us to understand the disease's diagnostic subtleties better. This understanding helped us optimize patient care, avoid overtreatment, and maintain a high quality of life.

## Conclusions

This case underscores the importance of a thorough and multidisciplinary approach in diagnosing head and neck masses, particularly when the clinical presentation is atypical. The complex nature of PTFL, with its histological features resembling other lymphoma subtypes such as classical follicular lymphoma, IRF4-rearranged large B-cell lymphoma, pediatric nodal marginal zone lymphoma, and reactive follicular hyperplasia, necessitates careful consideration and the use of comprehensive diagnostic tools. Clinicians must employ a combination of clinical assessment, imaging, molecular diagnostics, and immunohistochemistry to accurately differentiate PTFL from these other conditions. This detailed diagnostic process not only ensures that an accurate diagnosis is made but also guides the development of an appropriate treatment plan, avoiding unnecessary procedures and interventions. The successful surgical management of this patient, followed by a vigilant monitoring strategy, was key to the effectiveness of the treatment.
